# Single-Side Two-Location Spotlight Imaging for Building Based on MIMO Through-Wall-Radar

**DOI:** 10.3390/s16091441

**Published:** 2016-09-07

**Authors:** Yong Jia, Xiaoling Zhong, Jiangang Liu, Yong Guo

**Affiliations:** 1 College of Information Science and Technology, Chengdu University of Technology, Chengdu 610059, China; jiayong2014@cdut.edu.cn (Y.J.); guoy@cdut.edu.cn (Y.G.); 2 School of Electronic Engineering, University of Electronic Science and Technology of China, Chengdu 611731, China; jgliuweb@gmail.com

**Keywords:** through-wall-radar imaging, wall layout mapping, stationary target detection, spotlight imaging, MIMO

## Abstract

Through-wall-radar imaging is of interest for mapping the wall layout of buildings and for the detection of stationary targets within buildings. In this paper, we present an easy single-side two-location spotlight imaging method for both wall layout mapping and stationary target detection by utilizing multiple-input multiple-output (MIMO) through-wall-radar. Rather than imaging for building walls directly, the images of all building corners are generated to speculate wall layout indirectly by successively deploying the MIMO through-wall-radar at two appropriate locations on only one side of the building and then carrying out spotlight imaging with two different squint-views. In addition to the ease of implementation, the single-side two-location squint-view detection also has two other advantages for stationary target imaging. The first one is the fewer multi-path ghosts, and the second one is the smaller region of side-lobe interferences from the corner images in comparison to the wall images. Based on Computer Simulation Technology (CST) electromagnetic simulation software, we provide multiple sets of validation results where multiple binary panorama images with clear images of all corners and stationary targets are obtained by combining two single-location images with the use of incoherent additive fusion and two-dimensional cell-averaging constant-false-alarm-rate (2D CA-CFAR) detection.

## 1. Introduction

In the application of building imaging detection based on through-wall-radar, in addition to the moving human targets [1,2], the building wall layout and the stationary targets in the building (such as furniture, appliances and stationary humans) are the other two basic detection objects [3,4,5,6]. Because of the scattering difference and wall penetration attenuation, the echo intensity of the building’s walls is much higher than that of the stationary targets in a building. Therefore, different processing modes are desired to obtain the wall layout image and stationary target images.

With regard to the wall layout imaging, the effect from the weak echoes of the stationary targets in the building can be neglected. Due to the approximate mirror reflection in the wall surface, most of the existing methods obtain the echoes of all building walls (i.e., generate a panorama layout image with the images of all building walls) by two rounds of synthetic aperture multi-location detection along with two adjacent sides of the building from two separate vertical views with two-sided wall surfaces [7,8,9,10,11]. In this case, data collection and processing are complicated, and the building surroundings are required to satisfy the two-side synthetic aperture detection, which harms the feasibility of wall layout imaging. A multi-view fusion approach is presented to implement rough imaging for all building walls. It performs a long-path synthetic aperture detection with a vertical view and two squint-views along with one building side [12]. Nevertheless, the long-path pass and the obscure imaging quality are issues that must be noted.

The imaging of the stationary targets in a building is realizable if enough target echoes are collected along with one building side, whether the synthetic aperture multi-location detection or multiple-input multiple-output (MIMO) real aperture single-location detection [13,14,15,16] is used. As for the stationary targets, the wall echoes behave like strong clutters. The images of stationary targets, especially targets near walls, are smeared or even masked by the side-lobes of wall images [17,18,19,20]. The existing suppression methods of wall echoes, such as adaptive filtering [17], subspace projection [18,19] and CLEAN[20], suffer a severe performance decline under the condition of inhomogeneous walls. Moreover, there exist chaotic multi-path clutters in the enclosed building space, which result from the propagation interactions of target-to-wall (including other building structures, such as floors and ceilings, as well) and target-to-target and bring about the ghost interferences for the images of stationary targets [21,22,23,24,25,26,27,28,29,30]. With regard to the multi-paths associated with walls, the existing algorithms find the positions of multi-path ghosts in the image domain [21,22] or multi-path clutters in the raw data domain [23] and then remove the ghosts or clutters on the basis of the known positions and the ideal mirror reflection characteristic of the walls. However, in real applications, wall positions are unknown prior to analysis, and the assumption of ideal mirror reflection is not credible. Moreover, for [21,22], when there are multiple targets, the images of some targets are superposed with the ghosts of other targets resulting in improper removal of true targets. The suppression algorithms focusing on the multi-path ghosts from target-to-target interactions are developed in [24,25] based on multi-path aspect-dependent features and sub-aperture imaging strategies. For all types of multi-paths, the better suppression algorithm is proved to be the multi-view image fusion based on the distribution difference of multi-path ghosts in two single-view images, which are generated from the collected echoes at two adjacent sides of the building with two different views vertical with the two-side walls [26,27,28]. Nevertheless, just like the aforementioned wall layout imaging, the algorithm practicability is limited by the two-side detection, especially two-side synthetic aperture detection. Besides, the coherence factor is applied in [29] to suppress the low-coherence multi-path ghosts in through-wall-radar imaging while it is not suitable for the mode of synthetic aperture detection due to the fully-coherent-focusing property for each multi-path ghost [30].

In this paper, based on MIMO through-wall-radar, we present the single-side two-location spotlight imaging for the wall layout and stationary targets in a building. Two proper locations are selected on one side of the building to deploy the MIMO through-wall-radar successively in two different spotlight views oblique to the walls, acquiring the echoes of all building corners located at the junctions of every two adjacent walls and all stationary targets. At each location, the echoes of some of the building corners and all or some stationary targets are collected to form a single-location image, including the images of these corners and stationary targets. Corresponding to these two detection locations, two single-location images are obtained and then, through incoherent addition fusion and two-dimensional cell-averaging constant-false-alarm-rate (2D CA-CFAR) detection, combined into a panorama image consisting of the images of all building corners and all stationary targets. Instead of the wall images in the aforementioned wall layout imaging, based on the intensity difference with respect to the target images, the corner images can be extracted to speculate the layout of the building walls indirectly. For the proposed imaging method, the single-side two-location real aperture detection is easy to implement in the real building surroundings, and the multi-path clutters are reduced in the case of squint-view detection in comparison to the vertical-view detection. Moreover, compared with the wall images, the corner images are provided with a smaller distribution area, whose sidelobes interfere less with the images of stationary targets. The preliminary results based on Computer Simulation Technology (CST) electromagnetic simulation software are provided to validate the presented single-side two-location spotlight imaging method.

## 2. Single-Side Two-Location Spotlight Imaging Model

**Figure 1:**
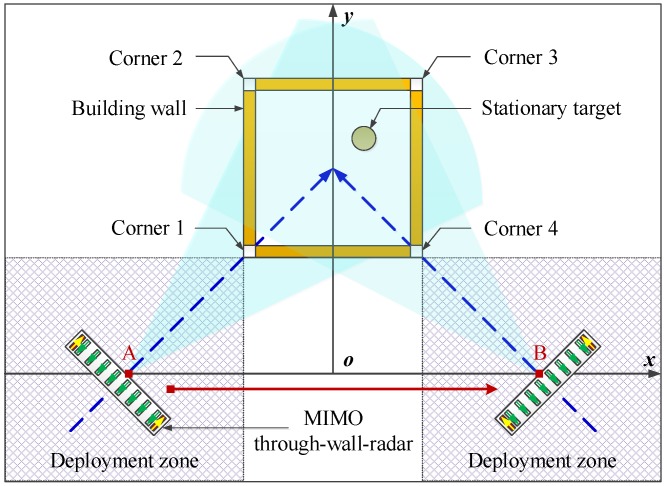
Single-side two-location spotlight imaging model based on multiple-input multiple-output (MIMO) through-wall-radar.

For simplification, a simple and typical building with four walls and four corners and a point target in the building are considered, as shown in Figure 1. Considering the approximate mirror reflection on the wall surface, it is difficult to collect the echoes of all building walls only along with one side of the building and then to generate the images of all walls for determining the wall layout directly. Fortunately, there exists a fixed relationship between the corners and walls of the building, namely the corner is located at the junction of two adjacent walls in general, and it is feasible to collect the echoes of all building corners only at one side of the building. Consequently, in this paper, instead of collecting wall echoes in the existing imaging method for wall layout, the echoes of all corners are desired to be acquired at one side and then used to generate the images of all corners, which can be employed to speculate about the wall layout.

In order to prevent complicated synthetic aperture detection and guarantee high azimuth resolution, the MIMO array is utilized for through-wall-radar real-aperture detection. Based on the consideration of the limited beam width, at one side of the building, at least two detection locations, such as A and B, are necessary to deploy the MIMO through-wall-radar in sequence in order to collect the echoes of all building corners and stationary targets along with two different spotlight views oblique to the walls. As operation guidance, we provide two deployment zones, as shown in Figure 1, where two detection locations can be selected appropriately according to the building structure and surroundings (one detection location versus one deployment zone). At each detection location, the echoes of a part of corners and all or partial stationary targets are obtained. For example, at Location A, the Corners 1 and 3 and the stationary target serve as the echoes, while the echoes of the Corners 2 and 4 coupled with the stationary target are collected at Location B.

**Figure 2:**
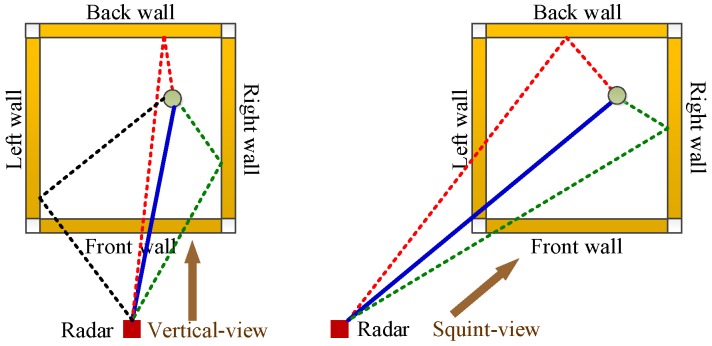
Multi-path propagation model under vertical-view detection and squint-view detection.

In addition to convenience, the squint-view detection also brings about two other advantages over the traditional vertical-view detection. The first one is fewer multi-path clutters, in other words fewer multi-path ghosts for the stationary target image. As shown in Figure 2, in order to simplify the analysis, only the first-order multi-paths associated with walls are considered by the dotted lines. It is clear that the squint-view detection makes the multi-path assigned to the left wall disappear compared with the vertical-view detection. In other words, the squint-view detection removes the multi-path of a one-sided wall. The second is that the formed corner images in the squint-view detection have much smaller distribution regions than the formed wall images in the vertical-view detection. Therefore, the sidelobe interferences on the stationary target image are reduced significantly by generating the corner images to substitute for the wall images. Moreover, considering the different squint-view detections in the two locations, the view dependence of multi-path ghosts and corner images makes it possible to reduce the ghosts and side-lobe interferences further through suitable fusion for two single-location images.

It should be mentioned that the squint-view detection gives rise to a decline of achievable spatial azimuth resolution in contrast to the vertical-view detection because of the larger range between the MIMO radar and a fixed target in the same rectangular coordinates.

## 3. Imaging Processing for Two-Location MIMO Radar Echoes

Assume the MIMO through-wall-radar in Figure 1 is operating with the ultra-wide-band pulse signal and *N* transmit-receive channels. Through the squint-view spotlight detection at Locations A and B successively, two groups of MIMO real-aperture echoes are collected as $\left\{S_{A}\left(n,t\right),n=1,2,\cdots ,N\right\}$ and $\left\{S_{B}\left(n,t\right),n=1,2,\cdots ,N\right\}$, which are utilized to generate two single-location images $I_{A}\left(X,Y\right)$ and $I_{B}\left(X,Y\right)$ based on the back-projection imaging algorithm as:

(1) $$I_{i}\left(x,y\right)=\sum_{n=1}^{N}S_{i}{\left(n,t\right)|}_{t=\tau_{i,n}},\;i=A,B$$

where $I_{i}\left(x,y\right)$ is the value of the pixel located at (x,y) in the single-location image $I_{i}\left(X,Y\right)$. $\tau_{A,n}$ and $\tau_{B,n}$ are the focusing delays defined as the propagation delays between the pixel at (x,y) and the transmit-receive antennas of the *n*-th channel at Locations A and B. From Array Positions A and B, the near corners, namely Corner 1 and Corner 4, consist of the free-space propagation condition, while the far corners (Corner 2 and Corner 3) and stationary target conform to the through-wall propagation situation. Consequently, the compensation of the wall effect is necessary to calculate the focusing delays accurately for the far corners and stationary target, but it is not required for the near corners. Under the conditions of known wall parameters or estimated wall parameters [31], the time-delay minimization approach in [32] can implement accurate wall compensation, but it suffers complex searching for four refraction points from the inside and outside surfaces of two adjacent walls in the case of squint-view detection. Therefore, in this paper, we employ the simple and approximate identical time-delay compensation method in [33] to calculate the focusing delays $\tau_{A,n}$ and $\tau_{B,n}$ as:

(2) $$\tau_{i,n}=\tau_{i,n}^{r}+2d\left(\sqrt{\varepsilon_{r}}-1\right)/c,\;i=A,B$$

where $\tau_{i,n}^{r}$ is the rectilinear free-space propagation delay between the transmit-receive antennas and the pixel, *d* is wall thickness, $\varepsilon_{r}$ is wall relative permittivity and *c* is the velocity of light. The time-delay offset $2d(\sqrt{\varepsilon_{r}}-1)/c$ is based on the assumption that the signal propagates through the wall always perpendicularly. Note that in order to perform wall compensation, the imaging scene needs to be divided into two sub-scenes where the near sub-scene only contains the near corners in free-space propagation and the far sub-scene just comprises the far corners and stationary target in through-wall propagation. The boundary of these two sub-scenes is set to the inside surface of front-wall which can be determined by the images of near corners. Therefore, we must first generate two single-location images without wall compensation and extract the strongest near corner images.

After the approximate wall compensation, in the single-location images $I_{A}\left(X,Y\right)$ and $I_{B}\left(X,Y\right)$, the far corner images and stationary target image will suffer from minor defocusing and displacement to an acceptable extent. Moreover, it is worth noting that the single-location image at Location A contains the images of the stationary target and the Corners 1 and 3, while at Location B, in addition to the image of the stationary target, the single-location image only consists of the images of the other corners, namely Corners 2 and 4.

With respect to these two single-location images including different corner images and the same stationary target image, image fusion is required to combine them into a high-quality panorama image with clear images of all corners and the stationary target. In order to ensure peer-to-peer contribution for image fusion, these two single-location images are normalized firstly as:

(3) $${\tilde{I}}_{i}\left(x,y\right)=\frac{I_{i}\left(x,y\right)}{max[abs\left(I_{i}\left(X,Y\right)\right)]},i=A,B$$

where ${\tilde{I}}_{i}\left(x,y\right)$ is a pixel value of the normalized single-location image ${\tilde{I}}_{i}\left(X,Y\right)$, abs(·) returns the absolute value and max(·) returns the maximum value.

Based on the complementary distribution characteristic of corner images, these two normalized single-location images, namely ${\tilde{I}}_{A}\left(X,Y\right)$ and ${\tilde{I}}_{B}\left(X,Y\right)$, are then combined into a panorama image $I_{+}\left(X,Y\right)$ with all corner images and the stationary target image through the incoherent additive fusion as:

(4) $$I_{+}\left(x,y\right)=\frac{abs\left({\tilde{I}}_{A}\left(x,y\right)\right)+abs\left({\tilde{I}}_{B}\left(x,y\right)\right)}{max[abs\left({\tilde{I}}_{A}\left(X,Y\right)\right)+abs\left({\tilde{I}}_{B}\left(X,Y\right)\right)]}$$

where $I_{+}\left(x,y\right)$ is the value of the pixel at (x,y) in the additive-fused panorama image $I_{+}\left(X,Y\right)$. It needs to be noticed that the incoherent rather than coherent additive fusion is chosen with respect to two stationary target images in these two single-location images ${\tilde{I}}_{A}\left(X,Y\right)$ and ${\tilde{I}}_{B}\left(X,Y\right)$. That is because without considering the wall effects on propagation, two stationary target images are non-coherent. This is caused by the different displacements from two squint-views.

Although the panorama image $I_{+}\left(X,Y\right)$ consists of the images of all corners and the stationary target, due to the range difference, scattering difference and wall penetration attenuation, there exists a significant intensity difference among these corner images and between the corner images and the stationary target image. Moreover, the multi-path ghosts and the side-lobes of the corner images have an adverse impact on the stationary target image, as well. In this case, it is difficult to observe the clear images of all corners and stationary target simultaneously from the panorama image $I_{+}\left(X,Y\right)$. Therefore, in this paper, we employ the 2D CA-CFAR detection for the image $I_{+}\left(X,Y\right)$ to generate a binary panorama image $I_{b}\left(X,Y\right)$ that is composed of the clear images of all corners and the stationary target with the same pixel value of one.

For the pixel at (x,y) of $I_{+}\left(X,Y\right)$, the test threshold of 2D CA-CFAR is given by:

(5) $$\beta \left(x,y\right)=\left[{\left(P_{fa}\right)}^{-1/M}-1\right]\sum_{j=1}^{M}I_{+}\left(x_{j},y_{j}\right)$$

where $P_{fa}$ is the constant-false-alarm probability, $I_{+}\left(x_{j},y_{j}\right)$ is the value of the reference pixel around the tested pixel at (x,y) and *M* is the number of all reference pixels. Besides, the discarded protection pixels need to be selected properly, confirming that all target pixels are not involved in the threshold calculation. Then, the test judgment for the pixel at (x,y) is performed as:

(6) $$I_{b}\left(x,y\right)=\left\{\begin{array}{c} \;1\;,\;I_{+}^{2}\left(x,y\right)>\beta (x,y)\\ 0\;,\;I_{+}^{2}\left(x,y\right)\leq \beta (x,y) \end{array}\right.$$

After implementing the test judgment for each pixel of $I_{+}\left(X,Y\right)$, we obtain a binary panorama image $I_{b}\left(X,Y\right)$ with the clear images of all corners and the stationary target. Nevertheless, it is difficult to identify the corners and target from the binary image without more information and then to reconstruct the wall layout and determine the target position within the building.

Consider that the intensity difference between the corner images and the stationary target image can be used to implement image attribute identification. We furthermore generate a reference panorama image $I_{r}\left(X,Y\right)$ maintaining the original images of all corners and the stationary target through the above 2D CA-CFAR detection processing with different outputs as:

(7) $$I_{r}\left(x,y\right)=\left\{\begin{array}{c} \;I_{+}\left(x,y\right)\;,\;I_{+}^{2}\left(x,y\right)>\beta (x,y)\\ \;0,\;I_{+}^{2}\left(x,y\right)\leq \beta (x,y) \end{array}\right.$$

In general, for a simple structure of a building, the corner images are coupled with higher intensity than the stationary target image. Therefore, based on the reference panorama image $I_{r}\left(X,Y\right)$, we can use a simple judgment strategy to extract all corner images from the corresponding binary panorama image $I_{b}\left(X,Y\right)$. With the help of a proper threshold, in the reference image $I_{r}\left(X,Y\right)$, the stationary target image can be marked when its maximum pixel value is smaller than the threshold, and the other images are identified as the corner images. Based on the one-to-one correspondence relationship, in $I_{b}\left(X,Y\right)$, the clear corner images and stationary target image can be divided and then be applied for speculating wall layout and determining target relative location within a building.

## 4. Preliminary Results Based on CST Electromagnetic Simulation

### 4.1. Simulation Setup

**Figure 3:**
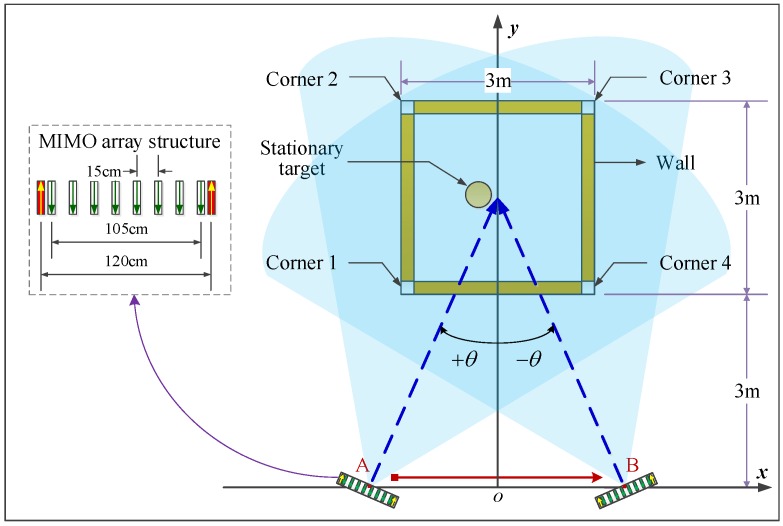
Single-side two-location spotlight imaging model in the CST simulation.

In this section, the CST electromagnetic simulation is introduced to demonstrate the proposed single-side two-location spotlight imaging method. As shown in Figure 3, a 3m×3m square building model is established including four walls and four corners with the same thickness of 10 cm and relative permittivity of six. At one side of the building, two locations are determined by the angle *θ* to deploy a two-transmitting and eight-receiving (2T8R) array successively, carrying out real-aperture spotlight detection with two different squint-views represented by +θ and -θ. In this simulation, the angle *θ* is set as $25^{\circ }$.

**Figure 4:**
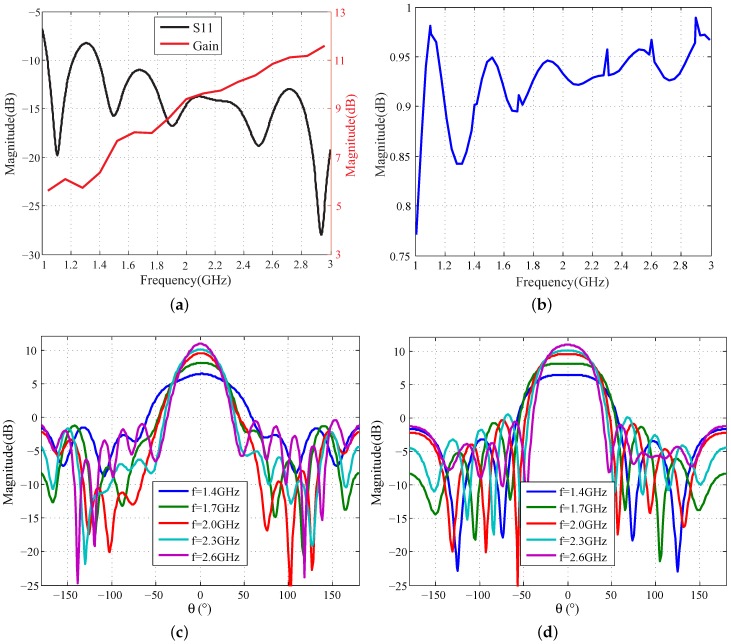
The characteristic curves of the simulated Vivaldi antenna: (**a**) Return loss and gain; (**b**) Radiation efficiency; (**c**) Beam pattern of the E plane; (**d**) Beam pattern of the H plane.

The 2T8R MIMO array is composed of 10 Vivaldi antennas where the spacing of two adjacent transmit-receive antennas is 7.5 cm and the inter-element spacing of two receiving elements is 15 cm. The 2T8R array is approximately equivalent to a uniform linear receiving array (ULRA) with 16 elements and the inter-element spacing of 15 cm, which makes the image side-lobes fall at a low level. The other basic characteristics of the Vivaldi antenna are shown in Figure 4. For the frequency band from 1 GHz to 3 GHz, which is suitable for wall penetration, the antenna gain reaches at least 6 dB, and the return loss and radiation efficiency are mostly less than -10 dB and more than ninety percent separately. Besides, the beam width of H plane is slightly greater than that of the E plane, so the H plane is selected to cover a larger area in the azimuth direction. It is also shown that the beam width decreases with the increasing of operation frequency. Moreover, at the higher frequency of 2.6 GHz, the 3-dB beam width of the H plane is about $52^{\circ }$.

**Figure 5:**
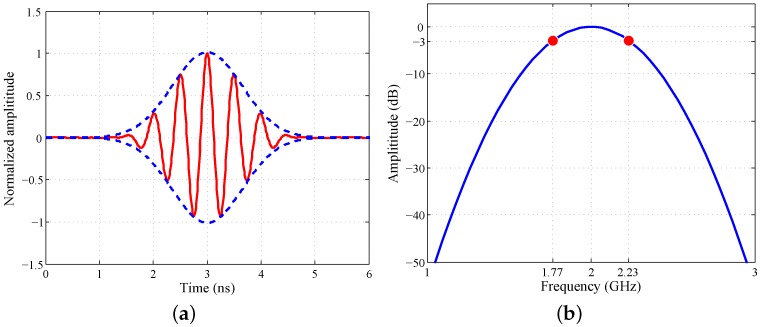
The waveform of the simulated actuating signal in (**a**) the time domain and (**b**) the frequency domain.

As shown in Figure 5, a sinusoidal signal with a Gaussian envelope is applied for the actuating signal. Its center frequency is 2 GHz, and the 3-dB bandwidth is about 460 MHz. Therefore, the range resolution is about 32.6 cm. Corresponding to the 3 dB of the frequency band of the sinusoidal signal, the 3-dB H plane beam width is about $65^{\circ }$, covering the whole building space in each location. For the center frequency of 2 GHz, the corresponding wavelength is 15 cm. Meanwhile, considering the equivalent aperture of 120 cm + 105 cm = 225 cm of the 2T8R array, the azimuth resolution is near 45 cm at the maximum range of about 7 m, namely the distance from the array position to the farthest corner. In addition, the 15-cm inter-element spacing of the equivalent ULRA equals the wavelength, rather than half of the wavelength, resulting in the image grating lobes. Fortunately, for this simulation, the image grating lobes appear outside the interested building scene, excluding the grating lobe interferences with the imaging quality.

### 4.2. Simulation Results and Analyses

**Figure 6:**
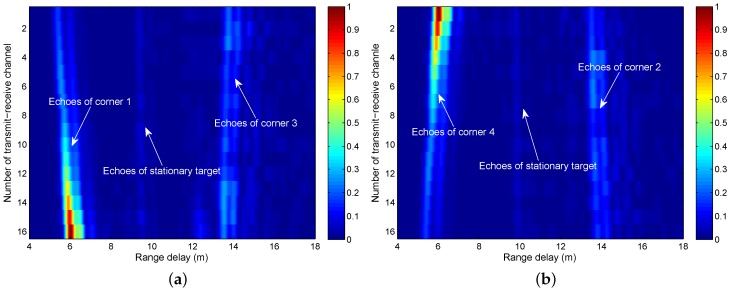
The range profile planes corresponding to (**a**) Location A and (**b**) Location B.

Firstly, we consider the case of a single stationary target in the building. A metal ball with a diameter of 30 cm is placed at the location of (x,y)=(-0.3m,4.5m). Through the two-location detection of the 2T8R array, two sets of 16-channel echoes are collected, as shown in Figure 6. It is clear that only two corners are observed in each set, while the stationary target appeared in both sets. Moreover, the echoes of corners, especially the near corners, are much stronger than the weak echoes of the stationary target. Along with the increasing of the channel number, the gradual change feature of the near corner echo intensity demonstrates that the corner exterior is subjected to mirror reflection, but the intensity-unchanging far corner echoes reveal that the corner interior represents a corner reflector.

**Figure 7:**
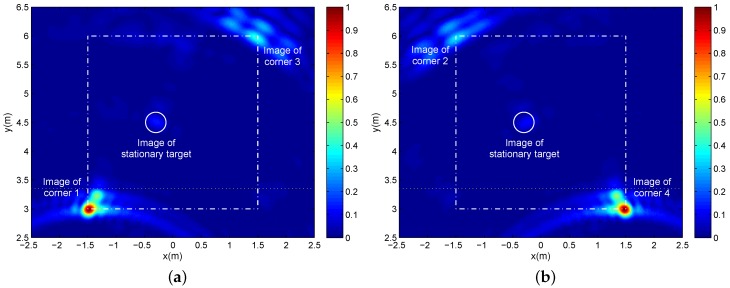
The single-location images corresponding to (**a**) Location A and (**b**) Location B, where the square dashed-dotted lines represent the preset wall layout, the circle represents the preset stationary target position and the dotted line represents the boundary of the near sub-scene and the far sub-scene.

Based on back-projection imaging and image normalization, two sets of 16-channel echoes are processed respectively to form two normalized single-location images in Figure 7 where the pixel resolution is 300×270 corresponding to the *x*-direction and *y*-direction, respectively. A different pair of all four corners is displayed in different single-location images, and the stationary target image appeared in both of the two single-location images. In each single-location image, only the near corner is displayed as a clear and accurate image, while the image of the far corner is blurred slightly. Besides, it is difficult to identify the weak image of the stationary target. The images of far corners and the stationary target almost focus on their real positions, which demonstrates the validity of the applied approximate wall compensation. In order to carry out wall compensation, we firstly generate two single-location images without compensating for the wall effect and then estimate the inside surface of the front wall according to the strongest near corner images. By employing the estimated inside surface as the boundary (the yellow or green dotted line in all imaging results), we divide the imaging scene into two sub-scenes, namely the near sub-scene only with the near corners in free-space propagation and the far sub-scene including the far corners and stationary target in through-wall propagation. Finally, the approximate wall compensation is accomplished only for the far sub-scene.

**Figure 8:**
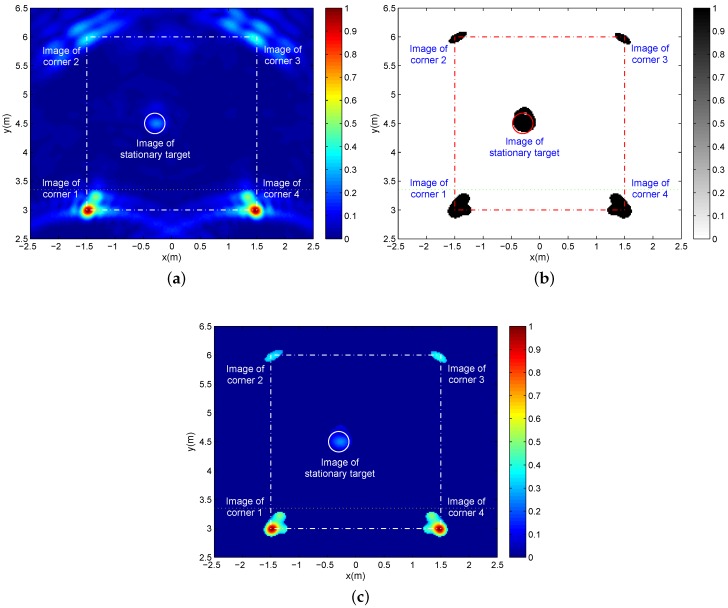
(**a**) The panorama image after additive fusion; (**b**) The binary panorama image after two-dimensional cell-averaging constant-false-alarm-rate (2D CA-CFAR) detection on (**a**); (**c**) The panorama image maintaining all corner and target images with the original pixel values after 2D CA-CFAR detection on (**a**).

With the images of the different corners and the same stationary target, these two single-location images in Figure 7 are combined into a panorama image with the images of all corners and the stationary target determined by the additive fusion. Figure 8a provides the additive-fused panorama image where multi-path ghosts are not apparent and the side-lobes of the corner images have almost no interference on the stationary target image. However, the significant intensity difference makes it hard to observe the images of all corners and the stationary target. After 2D CA-CFAR detection on the additive-fused panorama image with the constant-false-alarm probability of $P_{fa}=0.0005$ and the reference pixels in a 57×57 reference window without the interior 31×31 protection pixels, we obtain a binary panorama image as given in Figure 8b including clear images of all corners and the stationary target. In order to identify the corners and target from Figure 8b, we generate in Figure 8c a reference panorama image maintaining the original images of all corners and the target through the 2D CA-CFAR detection with the same parameters, but with different outputs of the original pixel values. It is clear that there is an apparent gap between the two maximum pixel values of target image and the weakest corner image (image of Corner 2). Therefore, based on the intensity difference of the corner and target images and a proper threshold, all corner images can be extracted from these two panorama images (Figure 8b,c) with a one-to-one correspondence relationship. Based on the relationship of building corners and walls, the clear images of each two adjacent corners in Figure 8b determine the position of a wall. Therefore, the images of all corners can be applied for determining the layout of all building walls, and then, the clear image of the stationary target in Figure 8b can be used to confirm its relative location in the building.

**Figure 9:**
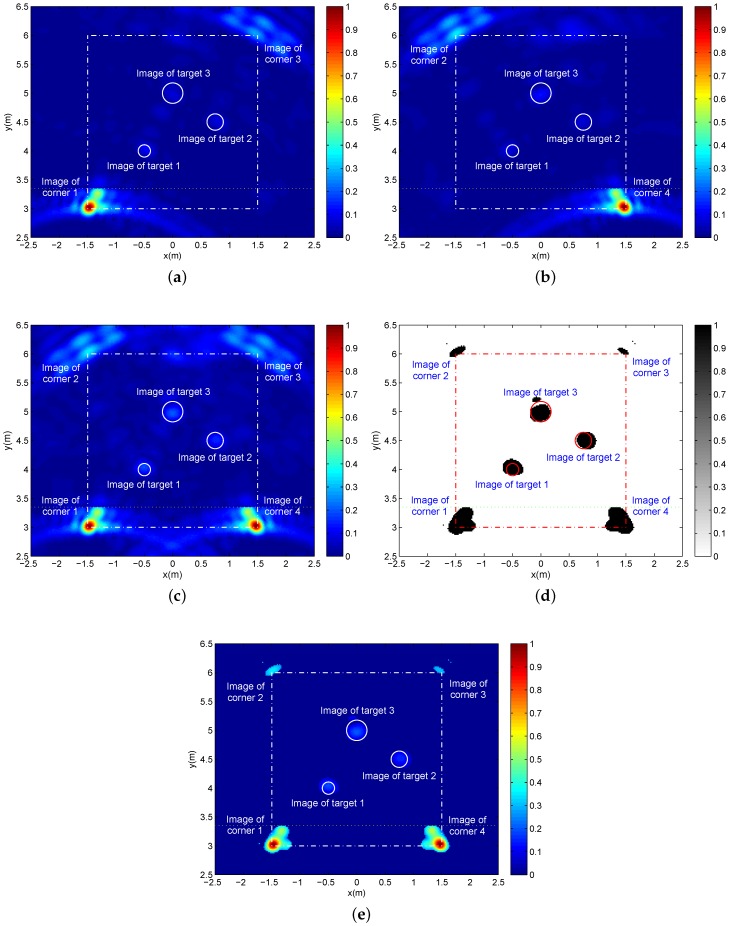
(**a**,**b**) Two single-location images corresponding to Location A and Location B, respectively; (**c**) The panorama image after additive fusion; (**d**) The binary panorama image after 2D CA-CFAR with $P_{fa}=0.001$, 61×61 reference window and 37×37 protection pixels; (**e**) The panorama image with the original pixel values after 2D CA-CFAR detection.

Then, we deploy three targets with different sizes at different locations to test the reliability of the proposed imaging approach. Target 1 is a 15 cm in diameter metal ball located at (x,y)=(-0.5m,4m). A metal ball of 20 cm in diameter is positioned at (x,y)=(0.75m,4.5m) as Target 2. Target 3 at (x,y)=(0m,5m) is a metal ball with a diameter of 30 cm. The other simulation parameters are maintained as the first simulation. From Figure 9a,b, we can find some ghosts derived from the target-target and target-wall multi-path clutters, which makes it difficult to observe the weak target images. Due to the view dependence of multi-path ghosts, the target images are endowed with a higher signal-to-clutter (ghost) ratio through the additive fusion, as shown in Figure 9c. As a result, by using the 2D CA-CFAR detection, the images of four corners and three targets are extracted successfully in Figure 9d–e. As the aforementioned simulation for the single target, the clear intensity difference between the corner images and target images provides a pathway to distinguish the image attributes.

**Figure 10:**
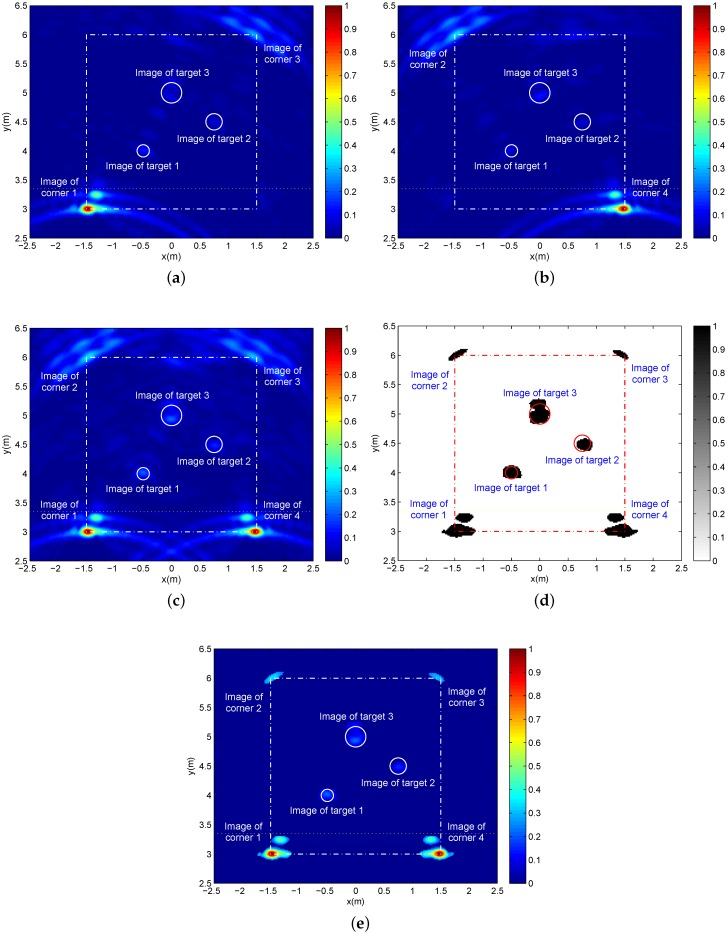
With the bandwidth of 770 MHz, (**a**,**b**) are two single-location images corresponding to Location A and Location B, respectively; (**c**) The panorama image after additive fusion; (**d**) The binary panorama image after 2D CA-CFAR with $P_{fa}=0.0002$, 61×61 reference window and 37×37 protection pixels; (**e**) The panorama image with the original pixel values after 2D CA-CFAR detection.

**Figure 11:**
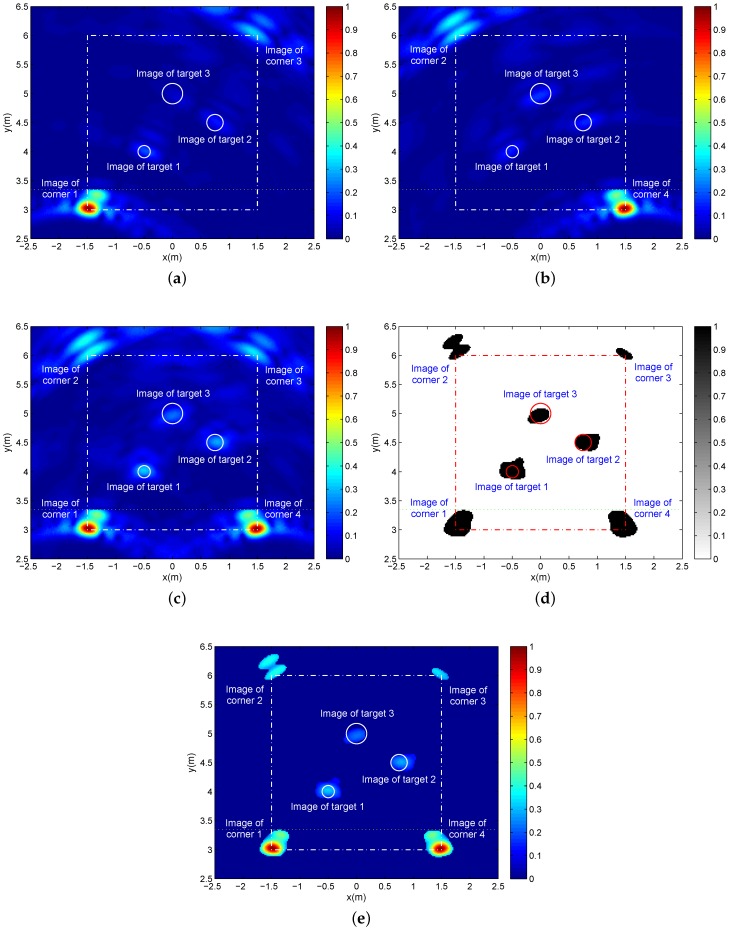
With the two-transmitting six-receiving (2T6R) array of 72 cm in length, (**a**,**b**) are two single-location images corresponding to Location A and Location B, respectively; (**c**) The panorama image after additive fusion; (**d**) The binary panorama image after 2D CA-CFAR with $P_{fa}=0.0005$, 61×61 reference window and 37×37 protection pixels; (**e**) The panorama image with the original pixel values after 2D CA-CFAR detection.

Considering that imaging quality is mainly determined by the range resolution and azimuth resolution, which are dependent on the signal bandwidth and array length, respectively, finally, we provide two groups of simulation results in Figure 10 with larger bandwidth (3 dB of bandwidth is about 770 MHz) and Figure 11 with a smaller array length and fewer array elements (the two-transmitting six-receiving (2T6R) array length is 72 cm, where the spacing of two adjacent transmit-receive antennas is 6 cm and the inter-receiving-element spacing is 12 cm). The other parameters remain as the above simulation with three targets.

On the whole, the images of four corners and three targets are detected successfully for both cases, as shown in Figure 10d,e and Figure 11d,e. As we know, a higher bandwidth brings about a better range resolution, which obviously benefits distinguishing multiple nearby targets. Moreover, from the detailed view and comparing with Figure 9, we can find in Figure 10 another advantage caused by the better range resolution. That is to say, the image of each wall corner is divided into two parts, namely the images of the inside surface and the outside surface of wall corner, which is beneficial to determining the wall layout. Furthermore, the better range resolution makes multi-channel echoes accumulate at the smaller image regions, increasing the signal-to-clutter and noise ratio (SCNR). However, the smaller region accumulation is more sensitive to the residual errors of multi-channel focusing time-delays after the approximate wall compensation. For this reason, compared with Figure 9a–c, Figure 6a–c only possesses a slightly higher SCNR.

With respect to the 2T6R array and in comparison to the 2T8R array, the smaller array length means worse azimuth resolution. As a result, comparing Figure 9 and Figure 11, the latter holds a larger spread for all target and corner images due to the worse azimuth resolution, which makes it difficult to distinguish multiple adjacent targets. Besides, the fewer array elements and the worse azimuth resolution translates into less echo accumulation at the larger image regions, which results in the lower imaging SCNR. Beneficially, the smaller array is coupled with the smaller angle between incidence and reflection, which reduces the wall penetration attenuation by shortening the propagation path in the wall and enhances the echoes of spherical targets. Based on this point and with the focusing time-delay errors of the less channels, the imaging SCNR can be improved inversely. Under the influence of all of the above aspects, Figure 11a–c with 2T6R has a near or a litter higher SCNR when compared to Figure 9a–c with 2T8R.

## 5. Conclusions

With the use of MIMO through-wall-radar, a novel single-side two-location spotlight imaging method is proposed to detect the wall layout of buildings and stationary targets within buildings. Only at two proper locations on one side of the building can we deploy the MIMO through-wall-radar in sequence to perform two rounds of real-aperture squint-view spotlight detection conveniently. As a preliminary research, incoherent additive fusion is employed to combine two single-location images, and then, through 2D CA-CFAR detection, a binary panorama image consisting of the clear images of all building corners and the stationary target is formed. Based on the image intensity difference between all corners and the stationary target, the corner images are extracted for speculating the wall layout indirectly. This can be used to determine the relative location of the stationary target within the building. As a supplementary point, the presented imaging method with two different squint-views reduces the interferences on the stationary target image from the multi-path ghosts and the side-lobes of the corner images (compared with wall images).

In future work, based on the view dependence of multi-path ghosts and corner images in the two-location squint-view detection mode, we will develop better fusion algorithms for two single-location images to reduce the interferences further. Moreover, the experimental validations in multiple practical building environments must be accomplished to test and improve the present imaging method.

## Abbreviations

MIMO	multiple-input multiple-output
CST	Computer Simulation Technology
2D CA-CFAR	two-dimensional cell-averaging constant-false-alarm-rate
ULRA	uniform linear receiving array
2T8R	two-transmitting and eight-receiving
2T6R	two-transmitting and six-receiving
SCNR	signal-to-clutter and noise ratio
